# Stress-responsive transcription factor families are key components of the core abiotic stress response in maize

**DOI:** 10.1093/g3journal/jkaf223

**Published:** 2025-10-14

**Authors:** Anna C H Pardo, Jeremy D Pardo, Robert VanBuren

**Affiliations:** Department of Horticulture, Michigan State University, East Lansing, MI 48824, United States; Plant Resilience Institute, Michigan State University, East Lansing, MI 48824, United States; Plant Resilience Institute, Michigan State University, East Lansing, MI 48824, United States; Department of Plant Biology, Michigan State University, East Lansing, MI 48824, United States; Plant Resilience Institute, Michigan State University, East Lansing, MI 48824, United States; Department of Plant Biology, Michigan State University, East Lansing, MI 48824, United States; Department of Plant, Soil, and Microbial Sciences, Michigan State University, East Lansing, MI 48824, United States

**Keywords:** stress biology, gene expression, maize

## Abstract

Abiotic stresses, including drought, salt, heat, cold, flooding, and low nitrogen, are harmful to agriculture and increasing in frequency due to climate change. Plants can experience multiple stresses within a single season, which elicit shared or overlapping responses. We searched for core stress-responsive genes in maize across stressors through meta-analysis of public RNA-seq data. Using nearly 1,900 RNA-seq samples with both set operations and random forest classification, we identified a core set of 744 stress-responsive genes across the six stressors. These are enriched in transcription factors, including the stress-responsive families AP2/ERF-ERF, NAC, bZIP, HSF, and C2C2-CO-like. Co-expression network analysis demonstrated that core transcription factors are co-expressed with stress-specific genes, supporting their role in regulating both generalized and stress-specific responses. This provides a valuable resource for understanding stress tolerance mechanisms and guiding future efforts to enhance maize resilience under climate change.

## Teaser

To understand maize’s response to six abiotic stresses, Pardo *et al*. re-analyzed nearly 1,900 public transcriptome samples and found 744 core stress-responsive genes, as well as six stress-specific gene sets. The core gene set is enriched in stress-responsive transcription factors (TFs). Integration of the core genes with a gene regulatory network revealed that these core TFs are more likely to regulate core genes than other genes, implying that regulation of core genes is distinct from that of stress-specific genes. However, these TFs are co-expressed with stress-specific gene sets as well, revealing regulatory crosstalk between core and stress-specific responses.

## Introduction

Abiotic stresses including drought, salt, flooding, heat, cold, and low nitrogen can severely impact agricultural crops. Climate change has slowed the growth of agricultural productivity, and events like droughts and floods are projected to increase in frequency (Calvin et al. 2023). This may decrease crop productivity (Raza et al. 2019). Maize (*Zea mays*) is an important food, feed, and biofuel crop, and may experience many abiotic stressors of different types throughout its growing season. Understanding the molecular basis of abiotic stress response is important for improving maize stress tolerance and agricultural resilience under climate change.

Abiotic stresses challenge plants at the cellular and molecular levels. Most abiotic stressors, including cold, low nitrogen, drought, and flooding, inhibit photosynthesis (Singh and Thakur 2018; Li et al. 2019; Wu et al. 2019; Qi et al. 2021). This leads to buildup of reactive oxygen species, which can damage cell components including membranes. Antioxidants like glutathione, which scavenge excess reactive oxygen species, are instrumental in salt, drought, flooding, and cold responses (Aslam et al. 2021). In stresses with an osmotic component, including drought, salt, and cold, plants frequently accumulate osmolytes like sugars, amino acids including proline and glycine betaine, and polyamines; these protect against water loss and may also be antioxidants (Sharma et al. 2019). Many responses are mediated by phytohormones including ethylene, abscisic acid (ABA), and jasmonic acid (JA), and are similar across stressors. Ethylene and JA are noted as active regulators under various stress conditions, including heat, cold, drought, and salt among others (EL Sabagh et al. 2022).

Transcription factors (TFs) regulate stress responses in phytohormone-dependent and independent manners. TFs in the ABF subfamily of bZIPs are activated by signal transduction following ABA recognition; they then activate the expression of NAC and AP2/ERF TFs (Yoon et al. 2020). These regulate other stress-responsive genes (Mizoi et al. 2012; Nakashima et al. 2012; Shao et al. 2015), which impact the plant's response to stress. Given the similarity of responses to different stresses, it is reasonable to hypothesize the existence of a core stress response controlled by core stress-responsive genes.

Core stress responses have been studied using two approaches: gathering transcriptomic data from an experiment with multiple different stress treatments; or conducting a meta-analysis of previously published transcriptomic data. Meta-analyses or multistressor experiments have been conducted in cotton (Tahmasebi et al. 2019), rice (Cohen and Leach 2019), sesame (Dossa et al. 2019), *Brassica napus* (Zhang et al. 2019), and *Arabidopsis thaliana* (Shintani et al. 2024; Sanchez-Munoz et al. 2025), and maize (Li et al. 2017), among others. These meta-analyses often used a relatively limited number of studies, sometimes only one per stressor, which may limit the power of the meta-analysis. Furthermore, they re-analyzed data from at most 500 transcriptome samples. The largest of these, by Sanchez-Munoz et al. (2025), re-analyzed 500 samples from 23 studies, including both microarray and RNA-seq data.

Here, we re-analyzed nearly 1,900 RNA-sequencing (RNA-seq) samples from 39 maize stress experiments, spanning a variety of genotypes, growth environments, tissues, and developmental stages. We leveraged these data to identify core genes using both a standard set operations approach, which classified genes as up- or downregulated across stress conditions, and a random forest classification approach, which selected genes with the highest feature importance. Using set operations, we also identified stress-specific genes. Analysis of core genes revealed their enrichment in several stress-related TF families, which were found to be co-expressed with other core genes and with stress-specific genes, indicating their possible role in regulating not only the core response, but stress-specific responses.

## Methods

### Curating maize RNA-seq data

This study utilized publicly available and previously published abiotic stress RNA-seq data in maize from the NCBI Sequence Read Archive (SRA). Only RNA-seq data that could be linked with published papers were used. Data were collected for drought, cold, heat, salt, flooding, and low nitrogen, which are the best studied abiotic stresses in maize. Polyethylene glycol and similar treatments such as sugar alcohols were not included in the drought data. BioProjects were only included in the study if they had at least one well-documented stress time point as well as either a control treatment or samples taken at experiment initiation. Thirty-nine BioProjects containing 1,981 samples total met these criteria, and were selected for use in this study (for details, see Supplementary Table 1) (Kakumanu et al. 2012; Ding et al. 2014; Campbell et al. 2015; Frey et al. 2015, 2020; Liu et al. 2015, 2020, 2022; Makarevitch et al. 2015; Divya Bhanu et al. 2016; He et al. 2016, 2019; Lunardon et al. 2016; Thatcher et al. 2016; Li et al. 2017, 2020, 2021; Mu et al. 2017, 2018; Song et al. 2017; Waters et al. 2017; Begcy et al. 2019; Danilevskaya et al. 2019; Jin et al. 2019; Wang et al. 2019a, 2019b, 2020; Yu et al. 2020; Zhao et al. 2019, 2021; Zhang et al. 2020, 2021, 2022; Guo et al. 2021; Yao 2021; Xu et al. 2022; Zhou et al. 2022; Friero et al. 2023; Kang et al. 2023; Pardo et al. 2023; Ying et al. 2023). Each of the six abiotic stresses had at least three independent experiments (BioProjects). The dataset includes stress-tolerant and sensitive maize genotypes, and hybrids, but not mutants or transgenic plants. Most samples were from leaf or other photosynthetic tissues, but roots, reproductive, and seed tissues were also included. Studies with any number of replicates were included.

### Processing the RNA-seq data

All data were downloaded from the SRA using the prefetch and fasterq-dump commands from sratoolkit v2.11.2 (https://github.com/ncbi/sra-tools). The raw RNA-seq reads were processed using Nextflow v23.04.1 and the nf-core rnaseq pipeline v3.11.1 (Di Tommaso et al. 2017; Ewels et al. 2020) (dependencies: https://github.com/nf-core/rnaseq/blob/master/CITATIONS.md). Within the pipeline, reads were trimmed using fastp (Chen et al. 2018) and transcripts were quantified using Salmon (Patro et al. 2017). Length-scaled TPM were then generated using tximport (Soneson et al. 2016). The exception to our processing workflow was a low nitrogen dataset (Ying et al. 2023; BioProject: PRJNA904734), which was previously analyzed and processed with STAR (Dobin et al. 2013). The convertCounts() function in the DGEobj.utils R package v1.0.6 (https://CRAN.R-project.org/package=DGEobj.utils) was used in R 4.2.1 (R Core Team 2022) to convert the provided count matrix into TPM.

All transcripts were pseudoaligned to the B73 v5 genome (Hufford et al. 2021). Our dataset contains a diversity of maize genotypes, including multiple with chromosome scale assemblies, and we initially processed the data by pseudoaligning each genotype to the corresponding reference genome when available. However, we ultimately decided to map all data to B73 for the following reasons: (i) mapping rates were comparable when pseudoaligning to B73 vs the corresponding genome for several inbred lines (see Supplementary Table 2); (ii) many genotypes do not have sequenced genomes or are hybrids; (iii) the B73 reference annotation is manually curated and more complete than others; and (iv) graph-based annotations and syntenic gene groupings are still incomplete, making it challenging to create an informative set of comparable genes across genotypes for downstream comparisons. Thus, given the goals of this study, and the above limitations, we chose to use a single genome for read mapping.

### Data exploration of experimental factors

Data exploration was conducted with principal component analysis (PCA) using the full dataset that includes 12 tissue types (referred to as “all tissues” hereafter), and for photosynthetic tissues only, which included leaf, leaf meristem, and shoot samples. The raw gene expression (transcripts per million—TPM) values were filtered to remove genes with zero variance across samples, and the TPM values were log2 + 1 transformed using numpy v1.24.3 (Harris et al. 2020 ). RNA-seq datasets were collected using different sequencing machines, read lengths, coverage, and under different experimental conditions, and we reduced the batch effect of BioProject using pyComBat v0.3.3 (Behdenna et al. 2023). PCA was run on the uncorrected and batch corrected data for the full dataset and photosynthetic data only, and the first two principal components were calculated using scikit-learn v1.2.2 (Pedregosa et al. 2011) and plotted using matplotlib v3.7.1 (Hunter 2007).

To further explore heterogeneity within the dataset, we modeled the first principal component (PC1) of log-transformed, nonbatch corrected TPM as a function of genotype, BioProject, treatment, and tissue, using the lm() function in R v4.2.1 (R Core Team 2022). Interaction effects were excluded for computational efficiency, and because interpreting the biological significance of interaction effects can be challenging. This same modeling approach was repeated for PC1 of batch corrected TPM, allowing us to compare results between the two models.

### Identification of stress-induced genes

Changes in gene expression between stressed and control samples were evaluated using fold change or the ratio of gene expression in stress-treated (T) and control, or nontreated (N) samples (TN-ratio; described in detail below) (Shintani et al. 2024). TN-ratio was calculated using the formula from (Shintani et al. 2024) as follows:


TN−ratio=(stress−treatedTPM+1)/(non−treatedTPM+1)

For our study, TN-ratio was calculated on a per-experiment basis where for a given BioProject, the mean TPM was calculated for each sample and/or replicate within the control or stress treated groups, and this was used to calculate TN-ratio.

We used criteria as outlined in (Shintani et al. 2024), where genes with a TN-ratio of greater than 2 were considered upregulated under the respective abiotic stress, and those with a TN-ratio of less than 0.5 were considered downregulated under stress. For set operations, we calculated the union of upregulated and downregulated genes for each experiment. The core genes shared across all six stressors were identified as the intersection of these sets, representing genes consistently differentially expressed under all stresses. Genes that were only differentially expressed for a subset or only one stress condition were also identified, and we refer to these sets as “stress-specific genes”. The overlap of up- and downregulated genes among experiments within each stressor was also examined.

This analysis and others subsequently described in this section were initially conducted both on all samples in the dataset and on samples from photosynthetic tissues only. However, while there were some differences in numbers of core and stress-specific genes between all tissues and photosynthetic tissues, there were few differences between tissue sets in the quality of random forest modeling (described below), and downstream analyses yielded results of interest only for all tissues. Thus, this paper focuses mainly on results found with all tissues.

### Hierarchical clustering of abiotic stresses

To determine the relationships of transcriptomic responses to different abiotic stresses, we performed hierarchical clustering. BioProject-corrected, log2 + 1 transformed TPM values were scaled to a *z*-score using scikit-learn. For each of the seven treatments (six stress conditions and control), a mean expression value of the scaled and transformed TPM data was calculated and used as input for hierarchical clustering and dendrogram visualization using scipy v1.10.1 (Virtanen et al. 2020).

### Random forest binary classification

Random forest models were used to classify whether samples were stressed or control. To avoid data leakage, all stressed and associated control samples for a single stressor were held out for use as the test set. Given the hypothesized existence of a core stress-response transcriptome, a random forest model tested on a stress it was not trained on was hypothesized to be able to accurately classify stressed and control samples. This was repeated for all stressors, so that each stressor was used as the test set once, resulting in a total of six models, each with separately tuned hyperparameters. Hyperparameters tuned for each model included bootstrapping, maximum tree depth, maximum features, minimum number of samples per leaf, minimum samples split, and number of estimators. In each iteration, all other samples were used for the training set.

The BioProject-corrected and log2-transformed TPM were used as features in the model such that each feature was a maize gene. SMOTE was used for upsampling to balance numbers of control vs stress using training data. As stated above, hyperparameters were tuned separately for each model, with individual models having different stressors as the test set. The optimal hyperparameters were then used for training and making predictions.

For each of the six models fit for each set of samples, feature importance was calculated for all features (genes) used in the model. For each model, evaluation of possible core gene sets was conducted via iterative feature selection, as follows. The corrected TPM were filtered to only the top *X* features, where *X* = 50, 100, 250, 500, 1,000, 1,500, 2,000, 2,500, 3,000, 4,000, 5,000, 6,000, 7,000, 8,000, 10,000, or 15,000. Following TPM subsetting, a new RF model was run on each subset and the model performance metrics accuracy, AUC, and F1 were calculated for each model. Based on the optimum model performance using the smallest subset of features, the top 6,000 most important features were extracted from each of the six individual stressor models. The intersection of these six sets of 6,000 genes each was calculated to get the core stress genes from random forest.

### Co-expression network analysis

A co-expression network was constructed with the corrected TPM of the full maize dataset using Weighted Gene Co-expression Network Analysis (WGCNA) (Langfelder and Horvath 2008, 2012). Briefly, WGCNA creates a correlation matrix comparing all genes in a dataset to each other. This matrix is then used to identify groups (“modules”) of genes whose expression is correlated, or in other words, genes that are co-expressed. In our analysis, a soft threshold of 9 was used for network construction. Hub genes, ie genes that had a high positive or negative correlation with most genes in the same module, were identified using the module membership generated with the signedKME() function from WGCNA. A threshold value of approximately 0.86, or the 95th percentile of the absolute values of module membership, was used as the cutoff for hub gene identification. Only genes with module membership >0.86 were considered hub genes. We then found which hub genes were found in each core gene set, and used Fisher's exact test implemented in Python using scipy.stats as described above, to test whether there were more core genes in the set of hub genes than expected by chance.

### Gene ontology term enrichment

Gene ontology (GO) term enrichment was performed with topGO v2.50.0 (Alexa and Rahnenfuhrer 2010) separately for Biological Process GO terms in upregulated and downregulated core genes from the set operations, random forest, and combined approaches, for both sets of tissues. We also ran GO enrichment for the stress-specific stress genes for each stressor, separately for upregulated and downregulated stress-specific sets, for each set of tissues, and for the genes in each co-expression module. Fisher's exact test was used as the enrichment test, and the classic algorithm was used. False discovery rate (FDR) *P*-value correction was used to adjust *P*-values, and an FDR adjusted *P*-value of less than 0.05 was considered statistically significant. GO terms were current as of 2024 March 25.

### Transcription factor enrichment

The list of TFs in the *Z. mays* B73 v5 genome was downloaded from Grassius (https://grassius.org/species/Maize, Yilmaz et al. 2009). We tested for enrichment of TFs from all families (not any particular family) in the upregulated and downregulated core genes using a one-sided Fisher's exact test implemented using scipy.stats v1.10.1 (Virtanen et al. 2020). We also tested for TF enrichment in the upregulated and downregulated stress-specific genes from each stressor. The “fdrcorrection” function from statsmodels.stats.multitest v0.13.5 (Seabold and Perktold 2010) was used to adjust the resulting *P*-values. As during GO enrichment, FDR-adjusted *P* values less than 0.05 were considered significant.

Again using information from Grassius, the sets of all upregulated core genes and all downregulated core genes for each tissue set, from both methods combined, were tested for enrichment of each TF family. TF families with FDR-adjusted *P* values of less than 0.05 were considered significantly enriched, while those with adjusted *P* values of between 0.1 and 0.05 were considered close to enriched and thus also selected for further analysis.

For the subset of core genes belonging to the enriched or near-enriched TF families, we identified what co-expression network module each gene belonged to. We then used Fisher's exact test via scipy.stats as above to determine whether modules containing these TFs of interest were enriched in core genes. Again, FDR was used to adjust *P* values and adjusted *P* of less than 0.05 was considered significant.

### Gene regulatory network construction and analysis

A gene regulatory network was constructed using the random forest method in GENIE3 (Huynh-Thu et al. 2010). The pyComBat-corrected, log transformed TPM data were used as input along with a list of known maize TFs from Grassius. Following network construction, we analyzed the differences in mean weight for target genes of core TFs from enriched families (identified above) in different gene sets, ie comparing core and stress-specific target genes to other target genes. This was done using Dunnett's *t* test implemented in the R package DescTools. To build a distribution of *P* values, Dunnett's *t* was repeated 5,000 times and *P* values for each comparison were saved. Results were considered statistically significant if the 97.5% confidence interval of the *P* value distribution was less than 0.05.

## Results

### Exploration of maize abiotic stress gene expression data

**Fig. 1.**
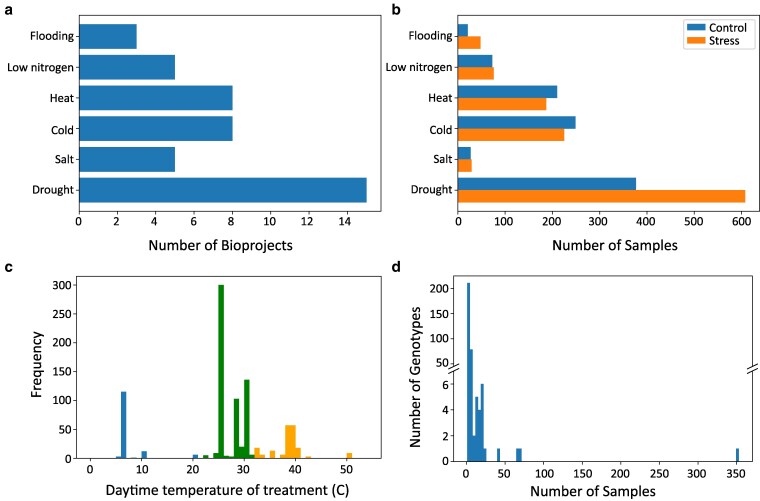
Summary of maize abiotic stress gene expression data. a) Number of BioProjects per stressor. b) Number of samples per stressor. c) Distribution of treatment temperatures, with cold (far left), heat (far right), and control (middle) showing distinct means but some overlap at the range edges. d) Distribution of sample numbers across genotypes, with most genotypes represented by few samples and only a few exceeding 50.

To search for conserved molecular signatures of abiotic stress responses, we gathered published maize RNA-seq data from the NCBI SRA. A total of 39 BioProjects were selected, including 15 studies on drought, 8 on heat, 8 on cold, 5 on low nitrogen, 5 on salt, and 3 on flooding (Fig. 1a). There were 1,872 total samples in the dataset with drought having the most samples and salt stress the fewest (Fig. 1b). Most samples were collected from leaf or root tissues, though a variety of other vegetative and reproductive tissues were also represented (Table 1). Experiments were conducted in greenhouse, field, and growth chamber environments, covering developmental stages from germination to reproduction. Treatment conditions varied across studies, particularly for temperature-based stress experiments. The temperature ranges defined as cold, heat, and control differed between studies, with the upper limits of cold treatments overlapping with the lower end of control conditions and a similar overlap occurring between heat treatments and control conditions (Fig. 1c). Additionally, the dataset spanned 328 maize genotypes, including both stress-sensitive and stress-tolerant lines, with inbred lines B73, W22, and Mo17 being the most frequently studied (Fig. 1d).

**Table 1.** Numbers of samples for each tissue type in the dataset.

Tissue	Number of samples
Leaf	1,394
Root	278
Shoot	46
Ear	44
Tassel	39
Embryo	24
Pollen	16
Stalk	10
Silk	9
Kernel	4
Ovary	4
Leaf meristem	4

All RNA-seq samples were downloaded from the SRA and re-analyzed using a common pipeline with the B73 V5 maize genome as the reference. Although substantial variation in gene content has been observed across maize diversity (Hufford et al. 2021), we used a single reference to enable comparisons across all datasets. We tested the impact of reference genome on read mapping rates for different genotypes. RNA-seq reads from the inbreds Oh43 and CML69 had comparable mapping rates when aligned to their corresponding de novo reference genomes and the B73 V5 reference genome (Supplementary Table 2). Overall, samples had an average read mapping rate of 76% with most having greater than 60% of reads mapped to B73 transcripts (Supplementary Fig. 1).

**Fig. 2.**
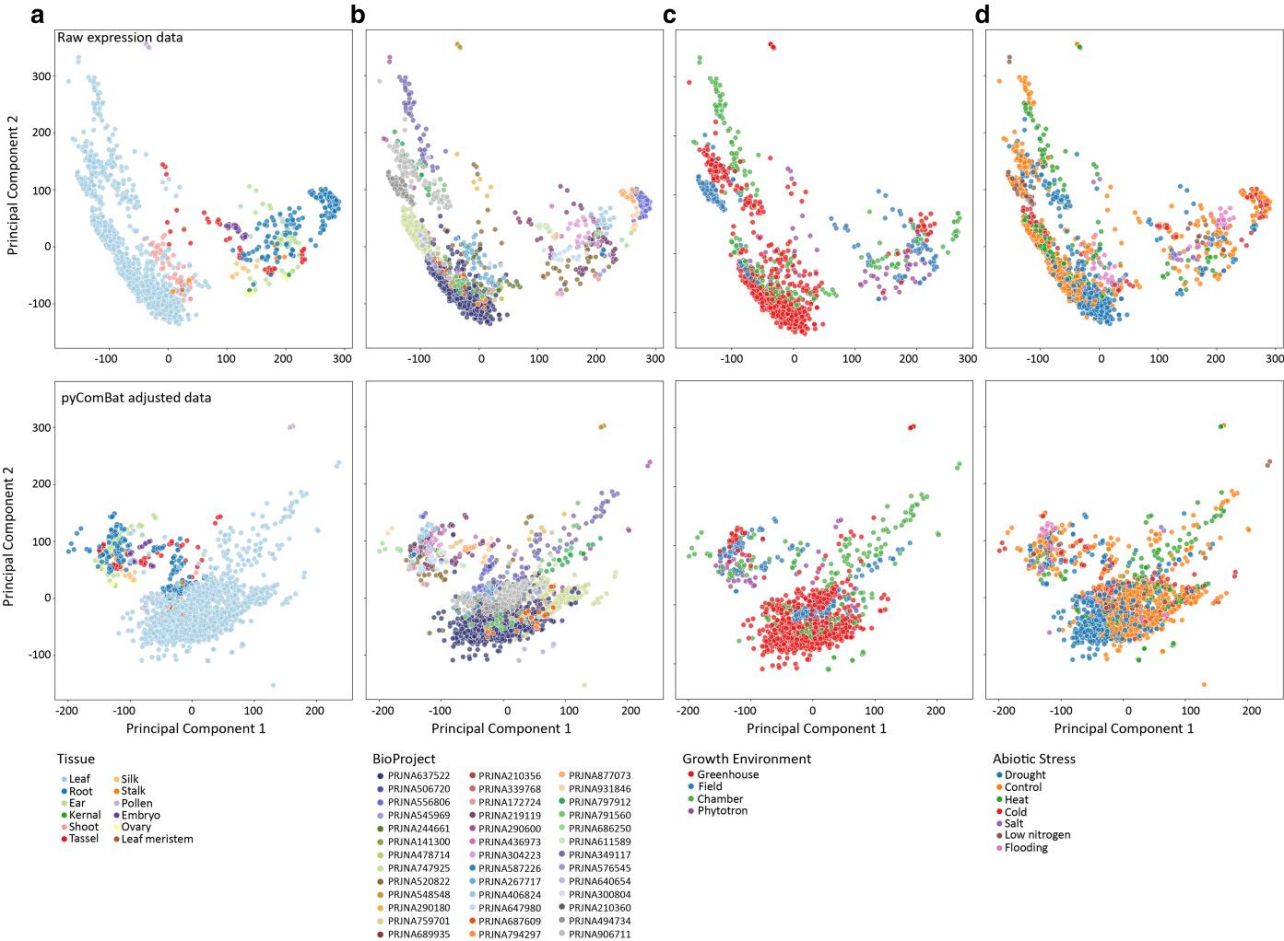
PCA of maize gene expression data. PCA biplots of log-transformed TPM values, shown without variance correction for BioProject effects (top) and after pyComBat correction (bottom). In the uncorrected data, PC1 explains 28% and PC2 explains 15% of the variance, while in the corrected data, PC1 explains 16% and PC2 explains 14%. Biplots are colored by tissue type (a), BioProject (b), growth environment (c), and treatment (d).

PCA was used to visualize sample separation. Approximately 28% of the variance in the full dataset was explained by PC1 and 15% by PC2. We found that samples grouped first by tissue type, with photosynthetic and nonphotosynthetic tissues segregating along PC1 and with separation within tissues primarily by BioProject (Fig. 2a and b). There was little apparent clustering of samples by growth environment (Fig. 2c), treatment (Fig. 2d), or developmental stage (Supplementary Fig. 2). Any clustering by these factors is likely an artifact of BioProject, as each experiment used different growth conditions, sampled at different developmental stages, and may have applied stress treatments differently.

We conducted linear modeling on the first principal component of gene expression, with genotype, BioProject, tissue, and treatment as independent variables. All four independent variables had a highly significant effect on gene expression (*P* < 0.001). Heterogeneity between genotypes, tissues, treatments, and experiments significantly contributes to the variability in gene expression among samples. Thus, we used pyComBat (Behdenna et al. 2023) to adjust for batch effects and reduce the variance due to BioProject, and re-ran the PCA with the corrected expression values (Fig. 2). For the pyComBat adjusted expression, PC1 explained about 16% of variance, and PC2 explained about 14%, and we observed an overall reduction in grouping due to BioProject (Fig. 2b). However, some grouping by BioProject was still evident, and this may be due to variability in other factors such as environment or genotype (Fig. 2c). We used this pyComBat adjusted expression matrix for all downstream analyses.

Following basic exploration of the dataset, we examined the relative similarity of transcriptomic responses to different abiotic stressors using hierarchical clustering on the batch corrected TPM. For each tissue set, hierarchical clustering identified two main clusters, but these differed substantially in composition between tissue sets (Supplementary Fig. 3), likely due to differences in tissue-specific abiotic stress responses.

### Identification and characterization of a core abiotic stress-responsive gene set

To identify the core stress-responsive genes across multiple abiotic stressors in maize, we used both set operations of the ratio of gene expression under stress vs normal conditions (fold change or TN-ratios; hereon, set operations) and the top predictive features of random forest-based machine learning models. Set operations is the typical method used for identification of core genes in meta-analyses (Cohen and Leach 2019; Dossa et al. 2019; Tahmasebi et al. 2019; Zhang et al. 2019; Shintani et al. 2024). To capture emergent expression patterns that would be missed from pairwise comparisons, we applied a random forest model to classify stressed samples. In this model, the most important predictive features (ie genes) that delineate stressed and healthy samples are defined as core genes. Support vector machine clustering was previously used to identify core stress-responsive genes in Arabidopsis (Sanchez-Munoz et al. 2025), but our random forest approach utilizes classification rather than clustering. Given our underlying hypothesis that a core stress transcriptome exists, we expected that a binary random forest classifier model would be able to predict whether a given transcriptome was from a stressed or control sample, even if the model had not been trained on the stressor on which it was being tested. This led to our “hold one stressor out” random forest approach (see methods for more details).

The efficacy of random forest prediction varied across stressors (Supplementary Fig. 4), although all area under the ROC curve (AUC) values were greater than 0.5. Salt and drought were consistently predicted most accurately, while low nitrogen, cold, and heat all had AUC values of slightly greater than 0.5. This is similar to the close clustering of temperature stressors with control after hierarchical clustering of treatments (Supplementary Fig. 3), which can further be explained by the fact that the low end of control temperatures overlaps with the high end of cold-treatment temperatures, and vice versa for heat (Fig. 1c). Thus, some “heat” and “cold”-treated plants may not have been fully physiologically stressed. These results indicate that our machine learning approach was able to capture biological patterns of responses to multiple abiotic stresses. The top features of the model are most predictive of stress vs control samples, and thus, would represent core stress responsive genes.

Using these combined methods, we identified a total of 744 core abiotic stress genes. Table 2 shows a summary of different categories of the core gene sets. Notably, more core genes were found via random forest than by set operations, and more core genes are upregulated than downregulated under stress conditions. The top features in the random forest models were generally differentially expressed under stress compared to control conditions, and there were only 11 core genes that were not differentially expressed.

**Table 2.** Summary of core gene numbers by different methods.

Method	Number of upregulated genes	Number of downregulated genes	Number of genes with no differential expression	Total genes
Set operations	193	66	0	255
Random forest	462	413	11	524
Combined	628	471	11	744

**Fig. 3.**
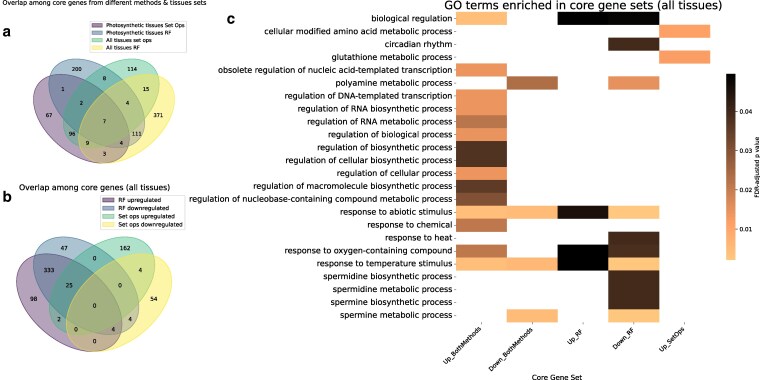
Basic characterization of the core abiotic stress gene sets. a) Overlap of core gene sets identified by set operations and random forest, as well as overlap between core genes from different tissue sets. b) Comparison of the overlap between upregulated and downregulated core genes (in any stress condition) for genes identified by random forest (“RF”) and set operations (“set ops”). c) GO enrichment analysis of core gene sets across tissues, highlighting terms related to regulatory processes, metabolites, and stress response.

Once core genes were identified, we tested for overlaps among these genes across these two approaches and whether they were up- or downregulated. We found that genes identified by the same method, whether by random forest or set operations, showed more similarity across different tissue sets than those identified by different methods within the same tissue set (Fig. 3a). For this reason, we focused primarily on core and stress-specific genes from all tissues for the rest of this study.

Core genes identified by random forest may be differentially expressed in opposite directions for individual stresses, and we observed a large overlap between upregulated and downregulated genes (Fig. 3b). This was not the case for core genes from set operations, where by definition the upregulated and downregulated genes were identified separately. There was minimal overlap between core genes identified from different methods (Fig. 3b), suggesting that the random forest core genes, identified based on their importance to the models rather than strictly by differential expression, may represent an emergent core response that would not be identified by set operations. Future core stress meta-analyses could benefit from a similar use of machine learning to identify emergent core stress genes rather than simple comparisons.

To investigate the functions of the core abiotic stress genes in maize, we ran GO term enrichment separately on the upregulated and downregulated sets of core genes from each method, including both methods combined. Several enriched terms were found for the core genes (Fig. 3c). These were largely distinct between upregulated and downregulated core genes, but four terms, “biological regulation”, “response to oxygen-containing compound”, “response to abiotic stimulus”, and “response to temperature stimulus”, were found across upregulated and downregulated core gene sets, albeit sometimes with different *P*-values (Fig. 3c). Generic responses to stimuli may take the form of either increases or decreases in abundance, and regulation may be applied by repression or activation.

Notably, GO terms specific to the downregulated core genes include various terms related to polyamine metabolism, especially that of the “higher” polyamines spermidine and spermine (Fig. 3c). Polyamines are stress-induced molecules that have protective roles in plants. While the detailed molecular mechanisms underlying this protection remain unclear, plants that overexpress polyamine biosynthetic genes often exhibit enhanced stress tolerance (Minocha et al. 2014; Bano et al. 2020). However, polyamine catabolism can also release reactive oxygen species, so it is possible that polyamines may also contribute to oxidative stress (Minocha et al. 2014). The GO term “circadian rhythm” was also uniquely enriched in downregulated core genes (Fig. 3c). Interactions between the circadian clock and abiotic stress responses are complex, but various clock components have been found to be downregulated under different stressors (Sharma et al. 2022). In upregulated core genes, specifically those found via set operations, other metabolism-related GO terms were enriched, including: “cellular modified amino acid metabolic process” and “glutathione metabolic process” (Fig. 3c). Amino acids play a crucial role in stress responses, including proline, which functions as a compatible solute (Batista-Silva et al. 2019). Glutathione is also a noted important antioxidant under various stressors (Aslam et al. 2021). In addition, many terms related to transcriptional regulation (ie “regulation of RNA biosynthetic process”) were enriched in the upregulated core genes, from both methods combined. This led us to investigate the presence of TFs among the core genes.

Stress-specific genes were defined as those that were differentially expressed in response to only one stress condition, in at least one study of that stressor, and were identified by set operations. The number of stress-specific genes varied by stressor (Table 3), with flooding consistently having the fewest stress-specific genes, likely because it also had the fewest experiments (ie BioProjects) in the dataset (Fig. 1a). Across tissue sets, heat had more upregulated than downregulated stress-specific genes, while drought and cold had more downregulated stress-specific genes (Table 3).

**Table 3.** Numbers of stress-specific genes for the six surveyed abiotic stresses in maize.

Set of tissues	Stressor	Number of upregulated genes	Number of downregulated genes	Total genes
All tissues	Cold	3,709	3,691	7,065
	Low nitrogen	1,355	1,487	2,825
	Heat	2,780	485	3,238
	Drought	1,599	3,609	5,092
	Flooding	390	254	643
	Salt	753	422	1,170

There are 11 GO terms that are enriched both in core genes from all tissues and stress-specific genes from all tissues. All of them are related to regulation, including “regulation of DNA-templated transcription”, “regulation of macromolecule biosynthetic process”, and “regulation of biosynthetic process”. These terms were enriched only in downregulated stress-specific genes from cold stress (Supplementary Fig. 5), while for core genes, they were enriched in upregulated core genes overall (Fig. 3c). Thus, it is possible that the cold stress response in maize is at least partially regulated by upregulated core stress genes.

### TF enrichment

**Fig. 4.**
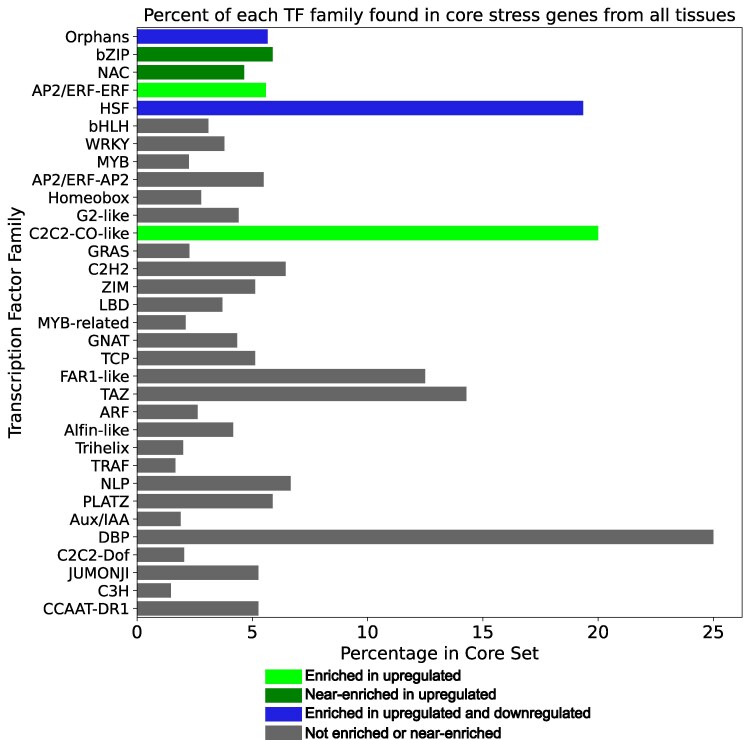
Distribution of TF families in core gene sets across tissues. TF families significantly enriched in upregulated core genes (*P* < 0.05, Fisher's exact test), near-enriched (0.05 < *P* < 0.1) in upregulated genes, or not enriched are highlighted.

We used Fisher's exact test to test for enrichment of TFs broadly and specific TF families in sets of core abiotic stress genes. In all cases, upregulated and downregulated core genes were tested separately. For general TF enrichment tests, core gene sets were also separated by method. General TF enrichment was found to be significant for the upregulated and downregulated genes both from RF and from both methods combined (all *P* < 0.001). Significantly enriched and near-enriched TF families were also found (Fig. 4, Table 4). With the exception of the orphan TF family, the enriched and near-enriched TF families have all been previously related to stress tolerance. These include the bZIP family, specifically the ABF subfamily, which is involved in ABA signaling (Yoon et al. 2020); the ethylene response factors (ERF) subfamily of AP2-ERF, which plays a role in flooding response (Mizoi et al. 2012); and the heat shock factors (HSFs), which regulate heat shock proteins (HSPs) (Andrási et al. 2021), important molecular chaperones responsive to various abiotic stressors (ul Haq et al. 2019).

**Table 4.** Summary of TF families enriched (adjusted *P* < 0.05) and near-enriched (0.05 < *P* < 0.1) in core gene sets.

Set of tissues	Regulatory direction	TF family	*P*-value
All tissues	Upregulated	AP2/ERF-ERF	0.0468
		C2C2-CO-like	0.0375
		HSF	0.005
		Orphans	0.005
		NAC	0.0924
		bZIP	0.0924
	Downregulated	HSF	0.0191
		Orphans	0.007

We also ran TF enrichment for the stress-specific genes, treating upregulated and downregulated genes separately. The following stress-specific gene sets were enriched in TFs: upregulated flooding-specific (*P* = 0.0422), upregulated cold-specific (*P* < 0.001), downregulated cold-specific (*P* < 0.001), and downregulated heat-specific (*P* < 0.001). Supplementary Table 3 shows the TF families that were enriched in different stress-specific gene sets. These were largely different from the families enriched in core genes, but there were some families enriched in both core and stress-specific sets. These included AP2/ERF-ERF, which was enriched in upregulated core genes and near-enriched in upregulated heat-specific genes; C2C2-CO-like, which was enriched in upregulated core genes and in upregulated flooding-specific genes; and NAC, which was near-enriched in upregulated core genes and enriched in upregulated heat, downregulated drought, and downregulated flooding-specific genes. ERF and NAC TFs, in particular, are noted for their stress responsiveness, so it is not surprising to find them enriched in both core and stress-specific gene sets.

### Co-expression network analysis of core abiotic stress genes

To further investigate the relationships among core and stress-specific genes and their potential regulatory roles, we constructed a co-expression network, enabling the identification of gene modules, regulatory hubs, and associations between TFs and stress-responsive genes. A co-expression network was generated from the batch corrected TPM for all samples using WGCNA (Langfelder and Horvath 2008, 2012). The co-expression network contained a total of 37,216 genes in 23 modules, with a mean module size of 1,618 genes. The largest module (M0) contained 10,959 genes and the smallest (M15) contained 103 genes (Supplementary Table 4).

**Fig. 5.**
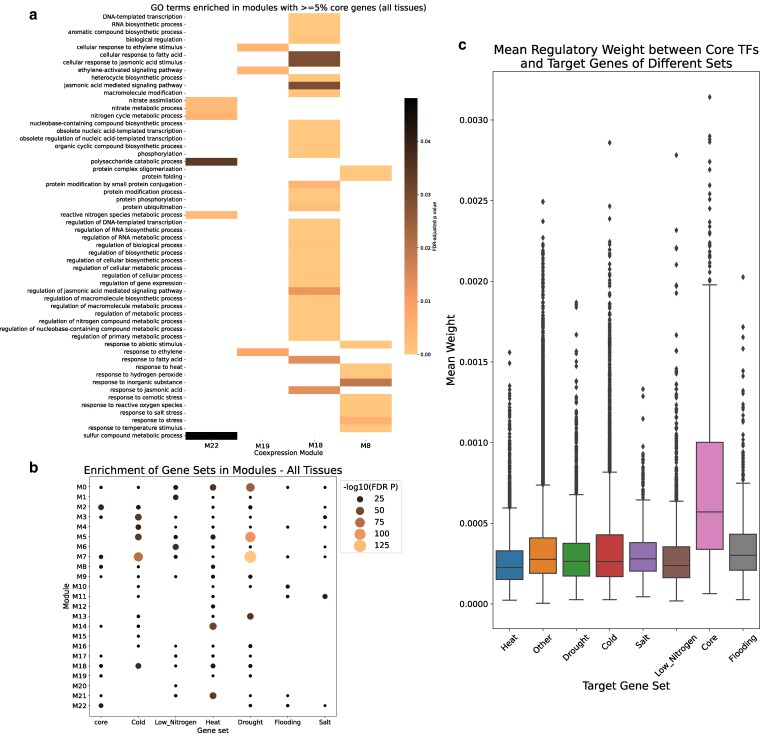
Co-expression module analysis of core and stress-specific gene sets. a) GO term enrichment analysis of modules containing at least 5% core genes. b) Bubble plots showing gene set enrichment in different co-expression modules for core and stress-specific gene sets. The negative log10 of the FDR-adjusted *P*-value is plotted, with larger bubbles indicating more significant enrichment; nonsignificant enrichments are not shown. c) Boxplot of regulatory link weights (mean per target gene) for 33 TFs, grouped by different gene sets. In (b), none of the modules enriched in core genes were exclusively enriched in core genes, as all were also enriched in at least one set of stress-specific genes; however, several modules were enriched in all six stress-specific gene sets. In (c), “other” represents genes not present in any of the core or stress-specific gene sets. Core TFs from enriched families were significantly more likely to regulate core genes than “other” genes.

Of the 23 co-expression modules, 21 had at least one core gene, and only the modules M16 and M13 contained no core genes. The five modules with greater than 5% core genes were M8 (9.6%), M2 (6.3%), M22 (6.2%), M18 (5.2%), and M19 (5.2%) (see Fig. 5a and b; Supplementary Table 4). We ran GO term enrichment for these modules to investigate their functions. Enriched GO terms were nonoverlapping for four of these five modules (Fig. 5a; there were no enriched GO terms found for module M2). Module M19 was associated with ethylene response, while module M22 was enriched for metabolic processes, particularly nitrogen metabolism. Module M18 had the highest number of enriched GO terms, many of which were related to regulation. Module M8 included terms related to various stimulus and stress responses, as well as protein folding, suggesting the presence of chaperones in this module. A total of 13 co-expression modules contained TFs previously identified within the core gene set, and eight of these modules were also enriched in non-TF core genes at *P* < 0.05 (Fisher's exact test).

Hub genes were identified from the co-expression data using the module membership correlation values, and genes with module membership above the 95th percentile were considered hub genes. Using these criteria, we identified 1,861 hub genes in the maize stress response network. Of these, 14 were core genes, which was not significantly more than expected by chance (Fisher's exact test, *P* = 0.999). None of the core hub genes were TFs. We also tested whether the co-expression modules were enriched in stress-specific genes for each stressor and in core genes using Fisher's exact test (Fig. 5b). In all modules where core genes were enriched, at least some sets of stress-specific genes were also enriched, indicating that core genes are not more strongly co-expressed with each other than with stress-specific genes. The M0, M22, M2, M3, M19, M8, M18, M6, and M5 modules all contained at least one core gene TF of one of the enriched or near-enriched families (Table 4), excluding orphans. All these modules except M5 and M6 were enriched in core genes in all tissues (Fig. 5b), and all were enriched in at least two stress-specific gene sets. This indicates that the core gene members of the AP2/ERF-ERF, NAC, bZIP, HSF, and C2C2-CO-like TF families are co-expressed with stress-specific genes and may regulate these genes.

### Regulatory network analysis of core stress genes

Based on the results of the co-expression network analysis, we hypothesized that core gene TFs from enriched families regulate not only other core genes but also stress-specific genes. To infer directional regulatory relationships beyond the correlations captured in co-expression networks, we constructed a gene regulatory network (GRN) using GENIE3 (Huynh-Thu et al. 2010). To assess the regulatory influence of core TFs, we used Dunnett's *t*-test to compare regulatory link weights between TFs from enriched and near-enriched families and various target gene sets. These included core genes, stress-specific genes for each stress condition, and a background gene set. For each comparison, we generated a distribution of 5,000 *P*-values from Dunnett's *t*-test and calculated the 97.5% confidence interval to determine significance.

We found that the core TFs of interest had significantly higher regulatory weights for other core genes compared to background genes (*P* = 0). In addition, Fig. 5c shows that the weights for core gene targets are higher than those for other targets; thus we can conclude that these important core TFs are significantly more likely to regulate core genes than non-core or stress-specific genes. Supplementary Table 5 shows the confidence intervals of *P*-value distributions for the stress-specific vs other targets comparisons. With a 97.5% confidence interval of 0.0039 to 0.0052, there is a significant difference between heat-specific and other target genes; there were no significant differences for any of the other gene set comparisons (Supplementary Table 5). Figure 5c reveals that on average, the weights for heat-specific target genes were lower than those of other targets. Thus, the important core TFs are significantly less likely to regulate heat-specific genes than the noncore or stress-specific genes.

## Discussion

Various abiotic stresses trigger similar responses, including oxidative stress, cellular damage, and metabolic disruptions. Plants express core stress-responsive genes to mitigate these challenges, along with unique pathways for each stress. Meta-analyses in various species have found 20 to 6,000 core stress-responsive genes based on set operations and, in one case, support vector machine clustering (Li et al. 2017; Cohen and Leach 2019; Dossa et al. 2019; Tahmasebi et al. 2019; Zhang et al. 2019; Shintani et al. 2024; Sanchez-Munoz et al. 2025). Previous efforts utilized relatively few stresses, microarrays with partial gene representation, or datasets that failed to capture the breadth of stress responses. We sought to expand on previous work using diverse genotypes, stresses, tissues, and stress severities in maize. Using both set operations and random forest, we found 744 core genes (Table 2), consistent with previous studies.

We leveraged RNA-seq data from 39 published experiments. Several previous meta-analyses analyzed data from fewer studies (less than 10 studies each in [Dossa et al. 2019] and [Cohen and Leach 2019]). While most previous studies used only set operations on differentially expressed genes, we broadened the core stress gene set by combining set operations with random forest. There was little overlap between the two methods’ results, indicating random forest detected emergent aspects of core stress response.

Expression changes under stress occur by action of TFs. We found the core stress genes were enriched in TFs, including families like HSFs, AP2/ERF-ERFs, C2C2-CO-like, NAC, and bZIP (see Table 4). These families have well-characterized roles in stress response (Mizoi et al. 2012; Yoon et al. 2020; Andrási et al. 2021). For instance, HSFs directly regulate HSPs (Andrási et al. 2021), which are molecular chaperones under various stresses (ul Haq et al. 2019). Core gene HSFs are present in a co-expression module with enriched “protein folding” GO terms, and are likely involved in regulating HSPs and thus protein stability under all abiotic stresses.

The AP2/ERF-ERF TFs respond to flooding, drought, and salt (Dong et al. 2012; Mizoi et al. 2012; Debbarma et al. 2019). The AP2/ERF-ERFs were enriched in upregulated core and heat-specific genes (Table 4), suggesting they are important regulators of these responses. Core gene AP2/ERF-ERFs are in a co-expression module whose only enriched GO terms are ethylene-related (Fig. 5a), highlighting potential ethylene regulation.

All TF families overrepresented in core genes were in modules that were also enriched in at least two stress-specific sets (Fig. 5b). Thus, these core TFs may regulate both the core and stress-specific responses. Gene regulatory network analysis reveals that while these TFs are significantly more likely to regulate core genes than genes that are neither core nor stress-specific, they do not show a similar pattern for any stress-specific gene set (Fig. 5c). These TFs are significantly less likely to regulate heat-specific genes vs other sets (Fig. 5c), indicating that, although crosstalk may exist between the regulation of core and stress-specific genes, distinct regulatory pathways likely govern different sets of genes.

These core TFs may be good stress tolerance improvement targets., Overexpression of stress-involved TFs has improved stress tolerance previously (Nakashima et al. 2012; Hoang et al. 2014; Baillo et al. 2019). However, further studies are required to elucidate the molecular mechanisms of the maize core stress genes. If a TF represses genes that are beneficial under stress, its overexpression could reduce stress tolerance. This meta-analysis does not provide enough information to say definitively whether the core genes would be good improvement targets; we hope follow-up studies will provide more information.

## Supplementary Material

jkaf223_Supplementary_Data

## Data Availability

All raw RNA-seq data used in this study is publicly available (see Supplementary Table 1 for details). Core and stress-specific gene information is provided in Supplementary File 1, and genes in co-expression modules are provided in Supplementary File 2. Other important data and code can be found at: https://github.com/achpardo/core-stress-transcriptome. Supplemental material available at G3 online.
