# Supplementary material for: Stress-responsive transcription factor families are key components of the core abiotic stress response in maize
Source: G3 (Bethesda). 2025 Oct 14;15(12):jkaf223. doi: 10.1093/g3journal/jkaf223 (PMC12693616; doi:10.1093/g3journal/jkaf223)
Supplement: jkaf223_Supplementary_Data [file jkaf223_supplementary_data.zip › Supplemental_Material_G3-2025-405883.docx]

SUPPLEMENTAL FIGURES AND TABLES


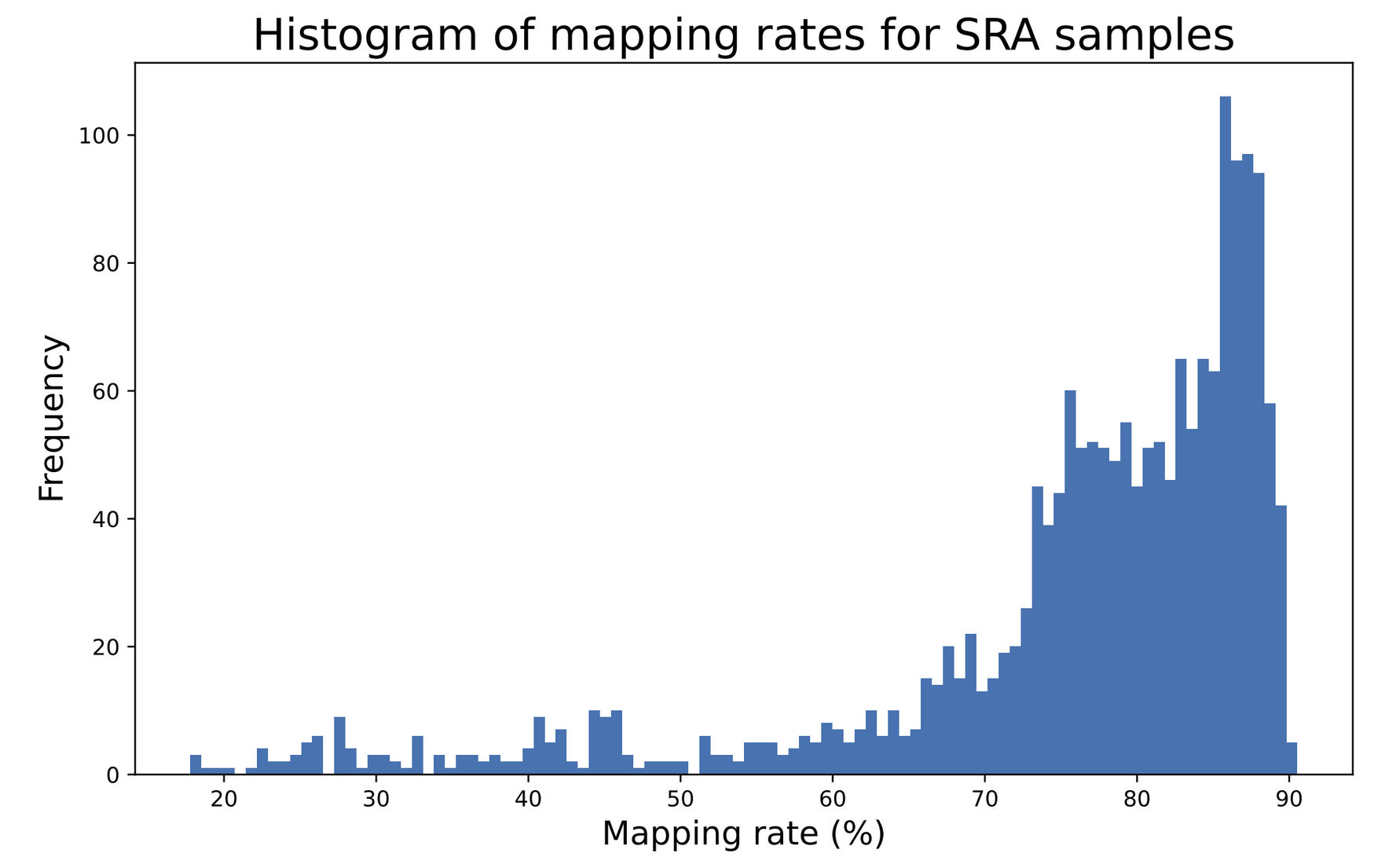


**Supplementary Figure 1: Salmon pseudoalignment mapping rates for all samples downloaded from the NCBI Sequence Read Archive and processed against the B73 V5 genome.**


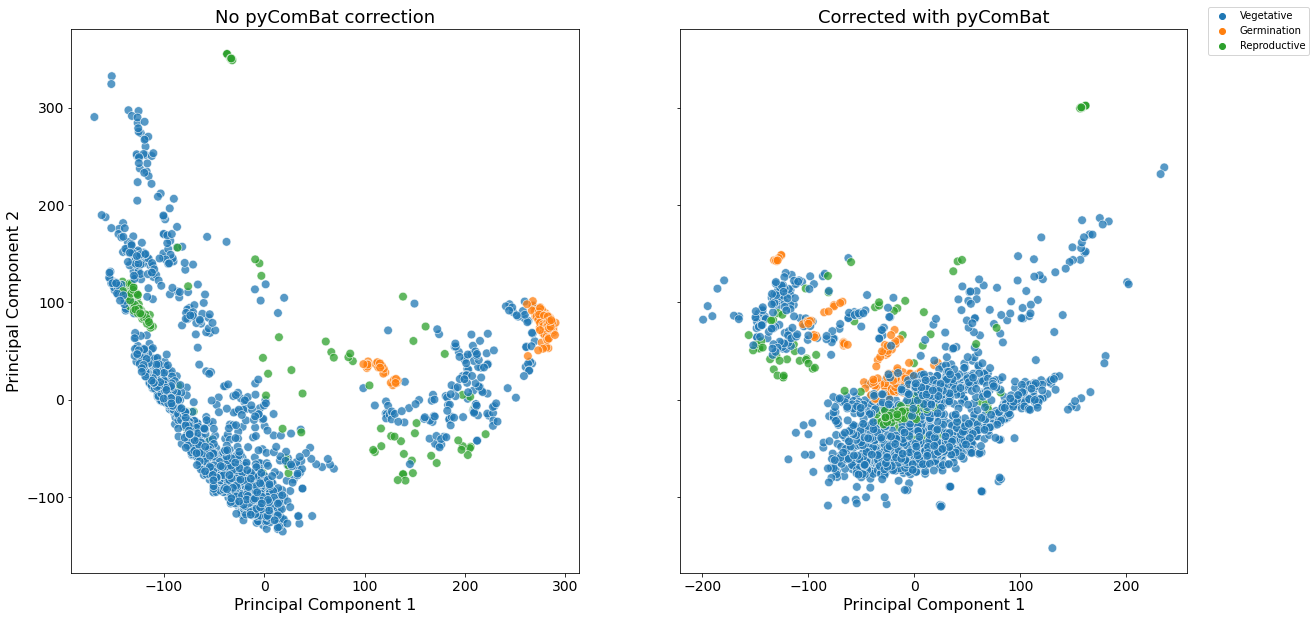


**Supplementary Figure 2: Principal component analysis biplot colored by developmental stage (vegetative, reproductive, or germination).** Uncorrected log(TPM) are on the left, corrected on the right.


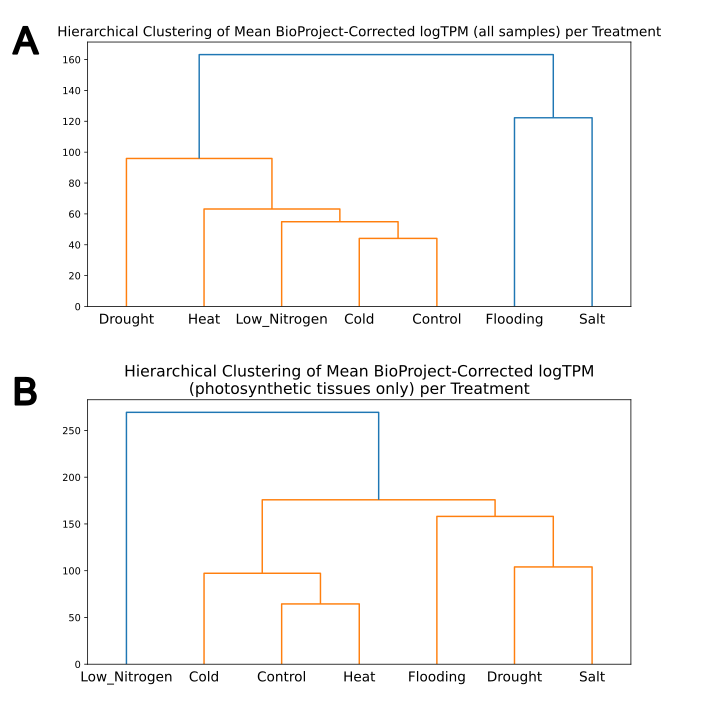


**Supplementary Figure 3: Hierarchical clustering of transcriptomes for different treatments, for all tissues (A) and photosynthetic tissues only (B).**


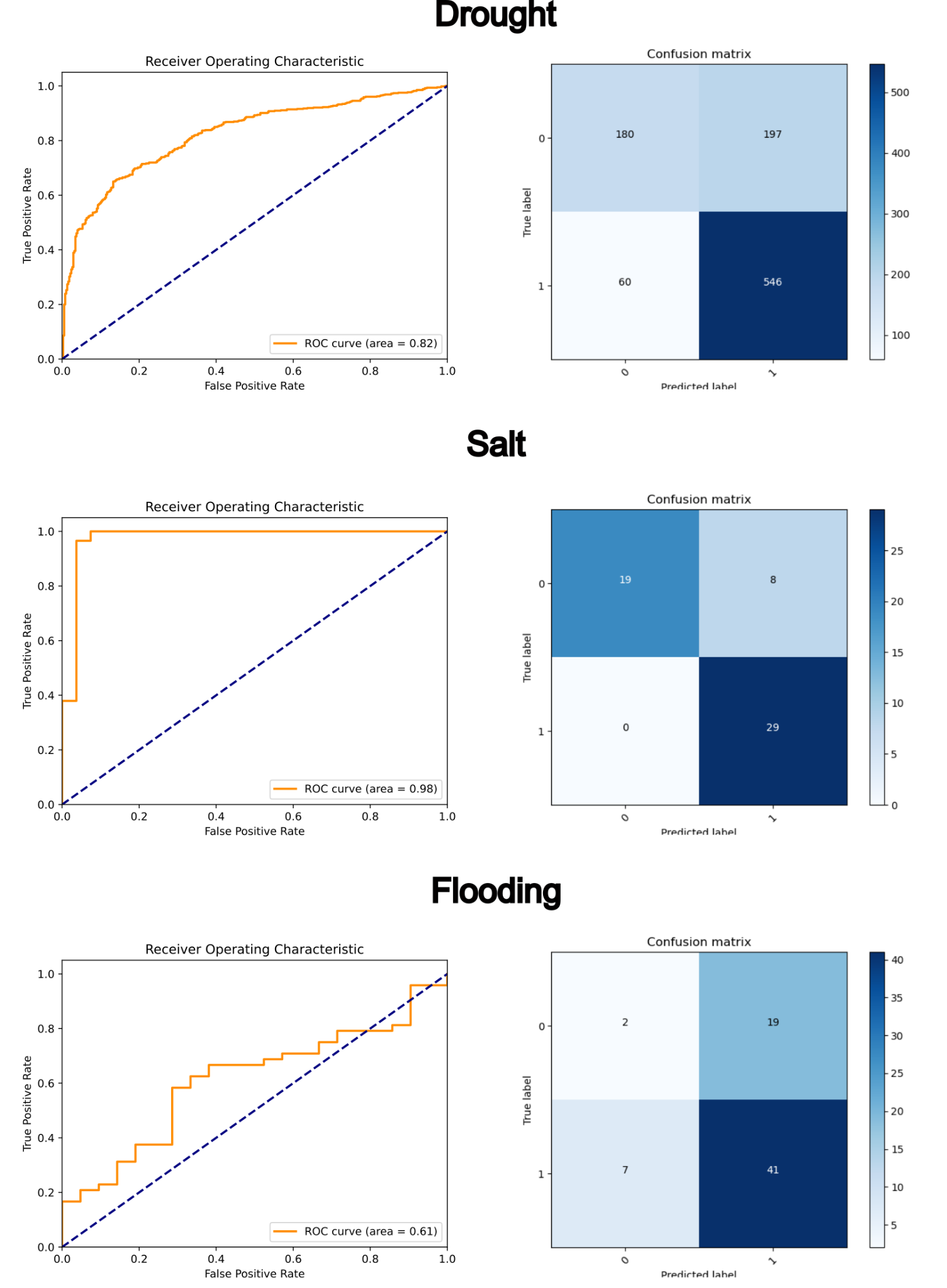


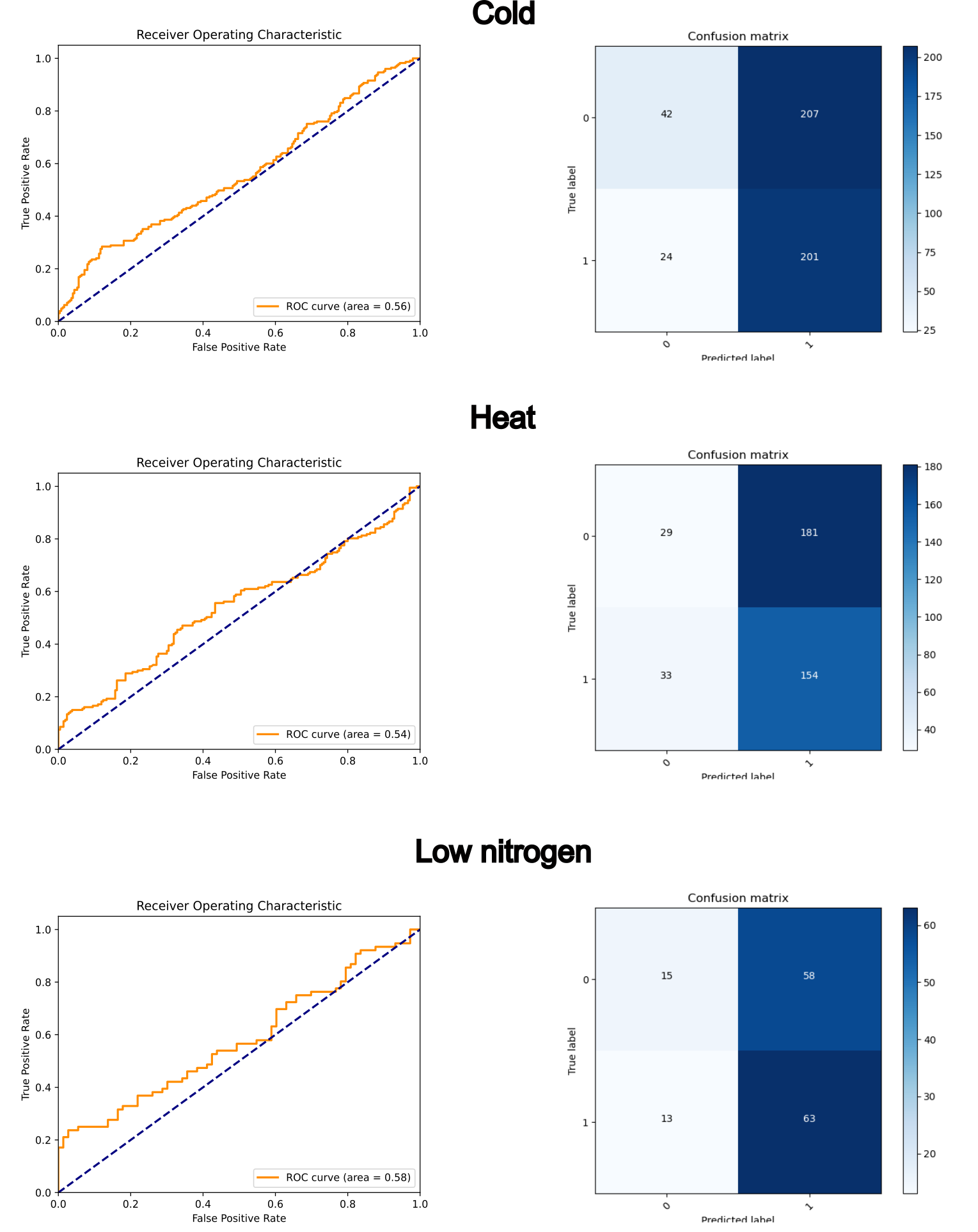


**Supplementary Figure 4: Model performance assessment for random forest classification.** Receiver operating characteristic (ROC) curves and confusion matrices for random forest classification models, with each stressor’s data held out as the test set.


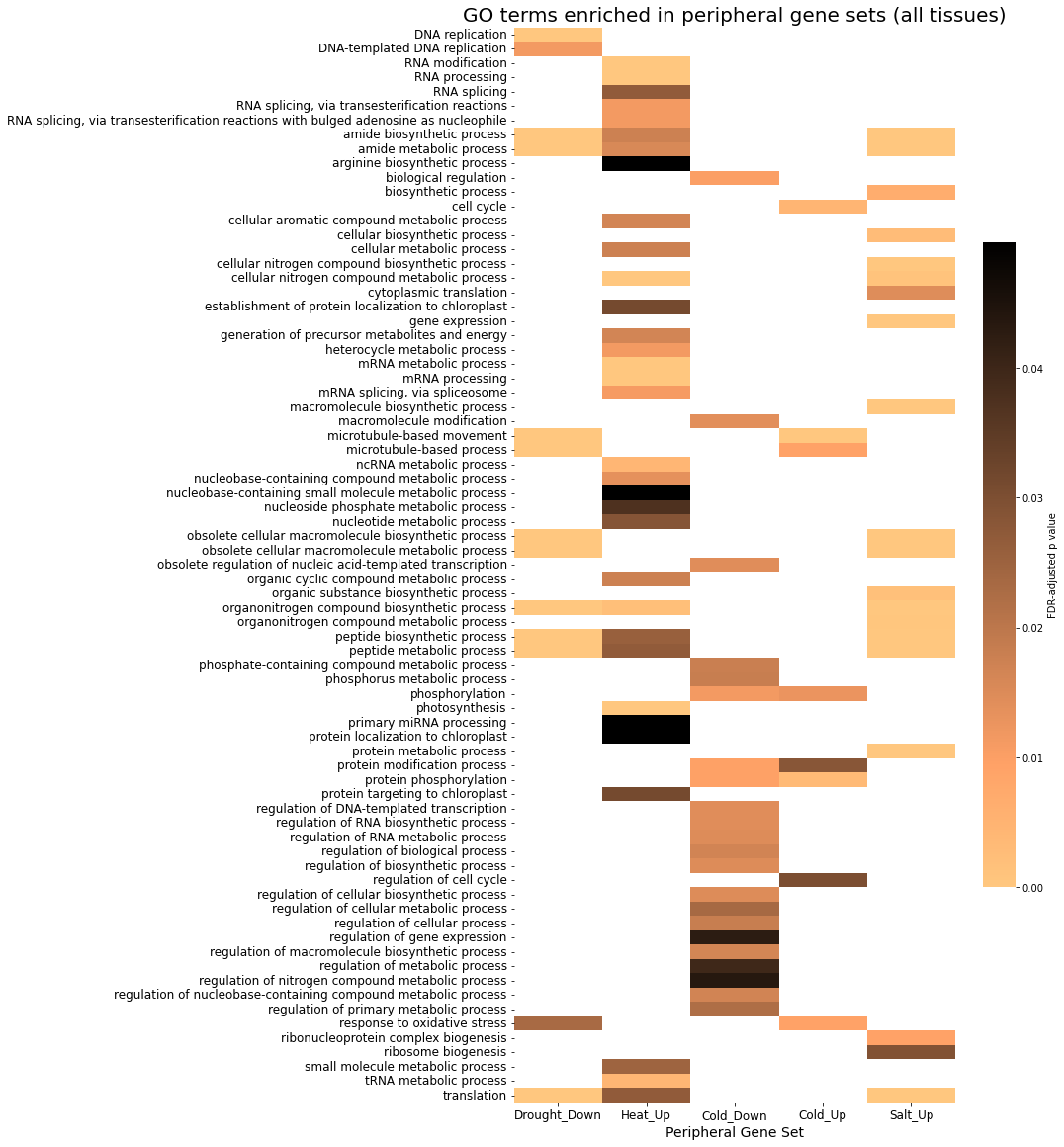


**Supplementary Figure 5: Enriched GO terms in stress-specific gene sets for all tissues.** Lighter colors indicate higher enrichment. Up, upregulated; Down, downregulated. Only the five stress-specific gene sets shown had any GO term enrichment.

**Supplementary Table 1: Description of published gene expression studies from maize.** Note, not all samples from each study were used in this meta-analysis; this table describes the metadata only for samples that were used.

| BioProject | Genotypes | Stressors | Developmental Stages | Tissues | Publication References |
| --- | --- | --- | --- | --- | --- |
| PRJNA637522 | 238, 268, 647, 1462, 4019, 5237, 7381, 8902, 05W002, 05WN230, 07KS4, 303WX, 4F1, 7884-4Ht, 835B, A188, B11, B113, B114, B73, By4944, By4960, By804, By809, By813, By855, CHANG72, CHUAN48-2, CI7, CIMBL1, CIMBL10, CIMBL101, CIMBL111, CIMBL114, CIMBL115, CIMBL12, CIMBL121, CIMBL122, CIMBL123, CIMBL125, CIMBL127, CIMBL129, CIMBL133, CIMBL139, CIMBL141, CIMBL142, CIMBL143, CIMBL144, CIMBL145, CIMBL149, CIMBL15, CIMBL150, CIMBL153, CIMBL157, CIMBL17, CIMBL18, CIMBL19, CIMBL2, CIMBL22, CIMBL23, CIMBL27, CIMBL28, CIMBL29, CIMBL32, CIMBL4, CIMBL40, CIMBL42, CIMBL46, CIMBL47, CIMBL51, CIMBL52, CIMBL53, CIMBL54, CIMBL55, CIMBL56, CIMBL58, CIMBL59, CIMBL6, CIMBL60, CIMBL62, CIMBL68, CIMBL69, CIMBL7, CIMBL70, CIMBL74, CIMBL75, CIMBL81, CIMBL82, CIMBL89, CIMBL9, CIMBL90, CIMBL91, CIMBL92, CIMBL94, CIMBL95, CIMBL96, CIMBL98, CIMBL99, CML115, CML116, CML118, CML121, CML130, CML134, CML162, CML163, CML165, CML170, CML171, CML172, CML189, CML192, CML20, CML298, CML304, CML31, CML32, CML325, CML360, CML361, CML411, CML423, CML431, CML432, CML433, CML454, CML470, CML479, CML480, CML493, CML496, CML50, CML69, D863F, DAN3130, Dan599, Dong237, FCD0602, GEMS10, GEMS11, GEMS13, GEMS14, GEMS2, GEMS20, GEMS21, GEMS28, GEMS30, GEMS32, GEMS36, GEMS37, GEMS39, GEMS4, GEMS40, GEMS41, GEMS42, GEMS44, GEMS46, GEMS5, GEMS54, GEMS55, GEMS58, GEMS6, GEMS60, GEMS63, GEMS65, GEMS66, GEMS9, Gy1032, Gy386, HuangC, Mo17, J4112, JH96C, Ji853, Jiao51, JY01, K10, LIAO138, LIAO159, LIAO5262, LY042, M153, MO113, Nan21-3, P178, PH4CV, PH6WC, R15X1141, Ry729, S22, SC55, Shen5003, SI273, SW92E114, Sy1032, Sy1039, Sy1052, Sy1128, Sy3073, Tian77, Tie7922, TY2, TY3, TY4, TY5, TY6, U8112, W138, W22, WH413, Wu109, X178, Ye52106, Ye8001, Yu374, ZB648, ZHENG35, Zheng58, Zheng653, ZHI41, ZHONG69, ZZ01 | Drought | V3 | Leaf | (Liu et al. 2020) |
| PRJNA506720 | An'nong 591 | Heat | Seedling | Leaf | (Zhao et al. 2019) |
| PRJNA556806 | B104, PE0002, PE0019, PE0022, PE0026, PE0040, PE0055, PE0075, PE0099, PE0100, PE0159, PE0161, PE0265, PE0323, PE0328, PE0334, PE0345, PE0349, PE0359, PE0420, PE0440, PE0485 | Cold | 4 days after sowing | Root | (Frey et al. 2020) |
| PRJNA545969 | B104 | Drought | V9, 5DAP | Ear, Leaf, Kernel | (B. Wang et al. 2019) |
| PRJNA244661 | B37, B73, Oh43 | Cold, Heat, Salt | 14 days old | Leaf | (Makarevitch et al. 2015; Waters et al. 2017) |
| PRJNA414300 | B46, NC236 | Salt | V1-V3 | Leaf | (M. Wang et al. 2019) |
| PRJNA378714 | B73 | Drought | V3 | Shoot | https://www.ncbi.nlm.nih.gov/bioproject/PRJNA378714/ |
| PRJNA747925 | B73, B73xMo17, B97, CML322, CML333, CML52, CML69, DK105, EP1, F7, Il14H, Ki11, Ki3, M162W, M37W, Mo17, Mo18W, Ms71, NC350, NC358, Oh43, Oh7B, P39, PH207, Tx303, W22, W22xB73, W22xMo17 | Cold, Heat | V2/V3 | Leaf | (Zhou et al. 2022) |
| PRJNA520822 | B73 | Heat | R1, V3 | Leaf, Tassel, Silk, Ear, Stalk, Root | (He et al. 2019) |
| PRJNA548548 | B73 | Heat | pollen maturation | Pollen | (Begcy et al. 2019) |
| PRJNA290180 | B73 | Drought, Salt | V5/V6 | Leaf | (Lunardon et al. 2016) |
| PRJNA759701 | B73, Mo17 | Cold | Germination | Embryo | (Xu et al. 2022) |
| PRJNA689935 | B73 | Drought, Cold | V3 | Leaf | (Guo et al. 2021) |
| PRJNA210356 | B73 | Drought | Two weeks old | Leaf | (Ding et al. 2014) |
| PRJNA339768 | B73 | Drought | VT | Leaf | (Song et al. 2017) |
| PRJNA172724 | B73 | Drought | 1 day after pollination | Ovary, Leaf meristem | (Kakumanu et al. 2012) |
| PRJNA291919 | B73 | Drought | V12, V14, V18, R1 | Leaf, Ear, Tassel | (Thatcher et al. 2016; Danilevskaya et al. 2019) |
| PRJNA420600 | B73 | Low Nitrogen | V6 | Leaf | (Mu et al. 2017) |
| PRJNA436973 | B73 | Low Nitrogen | V6 | Leaf | (Mu et al. 2018) |
| PRJNA304223 | B73 | Low Nitrogen | V2 | Root | (He et al. 2016) |
| PRJNA587226 | B73 | Low Nitrogen | Two weeks old | Root | (Wang et al. 2020) |
| PRJNA267717 | B73, B97, M162W, Mo18W | Flooding | V2 | Shoot | (Campbell et al. 2015) |
| PRJNA606824 | B73 | Flooding | Seedling | Root | (Yu et al. 2020) |
| PRJNA647980 | BML1234, L2010-3 | Salt | V2 | Root | (Zhang et al. 2021) |
| PRJNA687609 | Cim-3, Suwan-2 | Flooding | V8 | Leaf | (Yao 2021) |
| PRJNA794297 | CM37, cmh15 | Salt | V3 | Leaf | (Zhang et al. 2022) |
| PRJNA877073 | DKC 6664 | Cold | Seedling | Root | (Friero et al. 2023) |
| PRJNA913846 | FR697 | Drought | Seedling | Root | (Kang et al. 2023) |
| PRJNA379712 | H082183, Lv28 | Drought | Seedling | Leaf | (Li et al. 2017) |
| PRJNA791560 | H39_1, M189, ZD309 | Heat | 5 weeks old | Leaf | (Liu et al. 2022) |
| PRJNA686250 | He344 | Cold | 14 days old | Root | (Zhao et al. 2021) |
| PRJNA611589 | Ji853, Zao 8-3 | Cold | Germination | Embryo | (Zhang et al. 2020) |
| PRJNA349117 | L012, L017, L023, L043, P040, S058, S067, S070 | Heat | >=V4 | Leaf | (Frey et al. 2015) |
| PRJNA576545 | ND476, ZX978 | Drought | 45 days after germination | Leaf | (Jin et al. 2019) |
| PRJNA646054 | W22 | Heat | V4, V5 | Leaf | (Li et al. 2020; Li et al. 2021) |
| PRJNA300830 | Z59 | Drought | Reproductive stage, Mature Plant | Leaf | (Divya Bhanu et al. 2016) |
| PRJNA291064 | ZD619 | Drought | V2-V3 | Leaf, Stalk, Root | (Liu et al. 2015) |
| PRJNA904734 | B73xMo17, B73xPHN82, B73xPHZ51, LH195xPHN82, PHB47xPHN82 | Low_Nitrogen | V8, VT, R2 | Leaf | (Ying et al. 2023) |
| PRJNA906711 | CML69, Mo18W, Tx303, Ky21, CML52, B97, M37W, CML277, Ms71, Oh43, NC358, CML103, HP301, Tzi8, B73, CML247, Mo17, NC350, P39, Oh7B, CML322, Ki3, Ki11, Il14H, CML228, CML333, M162W | Drought | V5 | Leaf | (Pardo et al. 2023) |

**Supplementary Table 2: Salmon mapping rates of two genotypes of maize when mapped to their own de novo reference genomes and to the B73 v5 reference.**

| Genotype | Mean mapping rate against self | Mean mapping rate against B73 |
| --- | --- | --- |
| Oh43 | 89.08% | 85.45% |
| CML69 | 83.86% | 79.62% |

**Supplementary Table 3: TF families enriched in different stress-specific stress gene sets. P-values given are from Fisher’s exact test, corrected with FDR.**

| Set of tissues | Stressor | Regulatory direction | TF family | P-value |
| --- | --- | --- | --- | --- |
| All tissues | Cold | Upregulated | HMG | 0.0424 |
|  |  |  | OVATE | 0.0424 |
|  |  |  | SHI/STY (SRS) | 0.0094 |
|  |  | Downregulated | C2H2 | 0.0749 |
|  |  |  | WRKY | 0.0749 |
|  | Low nitrogen | Upregulated | CCAAT-HAP2 | 0.0619 |
|  |  |  | ZF-HD | 0.0234 |
|  | Heat | Upregulated | SWI/SNF-SWI3 | 0.0022 |
|  |  |  | mTERF | 0.0775 |
|  |  | Downregulated | ARF | <0.0001 |
|  |  |  | CCAAT-HAP2 | 0.0012 |
|  |  |  | SBP | 0.002 |
|  |  |  | Sigma70-like | 0.0661 |
|  | Drought | Upregulated | C2C2-YABBY | 0.0909 |
|  |  | Downregulated | CCAAT-DR1 | 0.0623 |
|  | Flooding | Upregulated | C2C2-CO-like | 0.0283 |
|  |  | Downregulated | MYB | 0.0087 |
|  |  |  | NAC | 0.0385 |

**Supplementary Table 4: Description of the number of total genes, core genes, and percentage of core genes found in each co-expression module.**

| **Module** | **Total number of genes** | **Number of core genes** | **Percent core genes** |
| --- | --- | --- | --- |
| M8 | 261 | 25 | 9.6% |
| M2 | 1762 | 111 | 6.3% |
| M22 | 1094 | 68 | 6.2% |
| M18 | 962 | 50 | 5.2% |
| M19 | 443 | 23 | 5.2% |
| M14 | 480 | 20 | 4.2% |
| M6 | 155 | 6 | 3.9% |
| M12 | 322 | 11 | 3.4% |
| M21 | 1068 | 34 | 3.2% |
| M4 | 185 | 5 | 2.7% |
| M5 | 2201 | 46 | 2.1% |
| M11 | 791 | 13 | 1.6% |
| M0 | 10959 | 158 | 1.4% |
| M20 | 1238 | 17 | 1.4% |
| M3 | 9813 | 130 | 1.3% |
| M1 | 159 | 2 | 1.3% |
| M15 | 103 | 1 | 1.0% |
| M10 | 260 | 2 | 0.8% |
| M9 | 738 | 5 | 0.7% |
| M7 | 3336 | 15 | 0.4% |
| M17 | 606 | 2 | 0.3% |
| M13 | 174 | 0 | 0.0% |
| M16 | 106 | 0 | 0.0% |

**Supplementary Table 5: Results of statistical analysis with Dunnett’s t test on gene regulatory network weights.** Each gene set listed here was compared to other target genes not belonging to any of those gene sets. Regulatory links were only considered between 33 transcription factors in the core gene set, belonging to enriched or near-enriched families (Figure 5), and target genes. Confidence intervals of 97.5% were calculated for distributions of 5,000 Dunnett’s t p values. Core genes are not shown; confidence intervals could not be calculated because all p values were 0.

| Gene Set | Lower confidence interval | Upper confidence interval |
| --- | --- | --- |
| Heat | 0.003935385 | 0.005192344 |
| Low nitrogen | 0.09833538 | 0.11006542 |
| Drought | 0.221322 | 0.2385571 |
| Salt | 0.3975272 | 0.4182547 |
| Flooding | 0.665957 | 0.6872534 |
| Cold | 0.8249245 | 0.8410558 |
